# Supplementary material for: Ultrastructural evidence of a mechanosensory function of scale organs (sensilla) in sea snakes (Hydrophiinae)
Source: R Soc Open Sci. 2019 Apr 10;6(4):182022. doi: 10.1098/rsos.182022 (PMC6502359; doi:10.1098/rsos.182022)
Supplement: Figure S1. Images of antibody controls on snake brain and skin [file rsos182022supp1.docx]

**Ultrastructural evidence of a mechanosensory function of scale ‘sensilla’ in sea snakes (Hydrophiinae)**

Jenna M. Crowe-Riddell, Ruth Williams, Lucille Chapuis, Kate L. Sanders

**Supplementary**

**
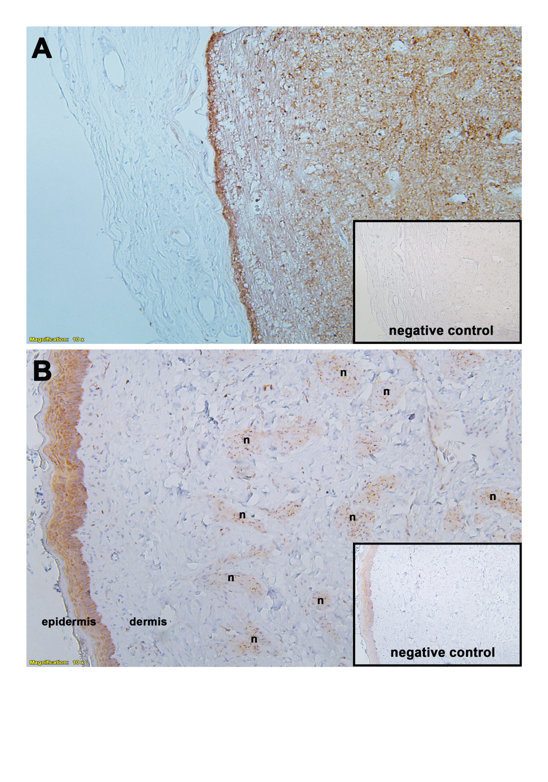
**

**Figure S1.** Primary and secondary antibody controls for PGP9.5 in A) taipan (*Oxyuranus scutellatus)* brain tissue and B) sea snake (*Hydrophis stokesii*) cephalic skin tissue. Negative controls were performed by omitting primary antibody incubation step. Note the generalised staining of the epidermis in snake skin, likely resulting from cross-reactivity of secondary and / or tertiary antibodies during immunohistochemistry procedure; nerve bundles (n) do not show same cross-reactivity.
